# Reply to Mathur et al.: Many-analyst studies should consider effect sizes and CIs

**DOI:** 10.1073/pnas.2219555120

**Published:** 2023-01-09

**Authors:** Nate Breznau, Eike Mark Rinke, Alexander Wuttke

**Affiliations:** ^a^Research Center on Inequality and Social Policy, University of Bremen 28334, Bremen, Germany; ^b^School of Politics and International Studies, University of Leeds, Leeds LS2 9JT, United Kingdom; ^c^Ludwigs Maximilian University, Geschwister Scholl Institute 80538, München, Germany

Mathur et al. (1) point out that the amount of variation we observed in our study (2) depends on how we look at the results. They suggest that the amount of variation in the findings of our teams appears more negligible from an effect size than from a statistical significance perspective. We agree completely that a focus on statistical significance as a measure of evidence may seriously impinge on researchers’ capacities to accurately assess evidence and that more could be done with our results in terms of effect sizes and CIs.

The importance of their observation is especially clear when we consider that a focus on statistical significance continues to be the standard practice across the social sciences. This is also evidenced in our study: Almost a third of the conclusions our research teams submitted (28.5%) held that the hypothesis was supported—despite the consistently small effect sizes. Support of this hypothesis by the team and the number of their models with a *P* < 0.05 and hypothesis-consistent direction correlate at 0.55 (0.66 with not achieving significance and rejecting the hypothesis). This suggests that statistical significance testing was a key aspect in the teams’ own conclusion formation processes. The rate of significant results per team explained 25% of the variation in subjective conclusions, the largest predictive variable by far. It is problematic that conclusions like those found in each team often become the headline findings of individual publications. Headlines often promoted reliant only on p values and without justification or recourse to the size and meaning of effects (3, 4). And while we should be cautious to automatically exclude “small” effect sizes as unimportant, in hindsight, as principal investigators who were responsible for writing up the results, we realize that we should have paid greater attention to effect sizes. We encourage readers to interpret the presented findings with this in mind.
